# Supplementary material for: Modeling impacts of faster productivity growth to inform the CGIAR initiative on Crops to End Hunger
Source: PLoS One. 2021 Apr 15;16(4):e0249994. doi: 10.1371/journal.pone.0249994 (PMC8049331; doi:10.1371/journal.pone.0249994)
Supplement: Attachment — Submitted filename: Response to reviewers - PONE-D-20-06988.docx [file pone.0249994.s001.docx]

**Response to reviewers of “Modeling impacts of faster crop productivity growth to inform the CGIAR initiative on Crops to End Hunger” [PONE-D-20-06988]**

by Keith Wiebe, Timothy B. Sulser, Shahnila Dunston, Mark W. Rosegrant, Keith Fuglie, Dirk Willenbockel, and Gerald C. Nelson

12 September 2020

**Authors’ responses** are highlighted in grey.

**Additional Editor Comments**:

Making assumptions explicit and explaining the rationale for making assumptions that have a direct impact om key outcomes is essential for any modeling exercise and is reiterated by the reviewers of the manuscript.

One way of addressing some of the concerns raised is adding through supplementary documentation the sensitivity analysis results related to contested parameter choices.

**Response**: We thank the editor and reviewers for their helpful comments, including identifying various points that were not clear. We have addressed these comments as explained in our point-by-point responses below.

It is also essential to make all the underlying data and model results publicly available. Not only is this a prerequisite for publication of a manuscript in PLOSone, this also facilitates peer review of results and helps identify inadvertent errors. The prepublication of the manuscript on SocArXiv [osf.io/preprints/socarxiv/h2g6r] brought a calculation error to the fore that you have since remedied.
Looking forward to the revised version of the manuscript addressing the important comments by the reviewers.

**Response**: We will make parameter values and model results available on GitHub prior to publication. We note that in addition to addressing comments raised during the PLOS ONE peer review process, this resubmission incorporates revisions that were made to address comments received on the prepublication version on SocArXiv after the original submission to PLOS ONE (as the editor indicates) – namely to correct an error in estimating the economic surplus for maize, to ensure use of a consistent set of prices throughout, and to report results for Nigeria as part of West Africa to avoid downscaling issues.

**Reviewer #1**:

This is a sound paper that addresses an important issue. It is ambitious and sometimes goes beyond the analytical capacity of the models.

Major comments
This is a sound analysis of an important problem—how to determine priorities among many CGIAR crops. The discussion covers a number of limitations of the analysis but there are others as well. Also quality of data need to be addressed. Finally, the conclusions should be sharpened. See details below.

**Response**: We thank the reviewer for this overall assessment. Specific comments are addressed below.

1. The high value of yams and bananas deserves more discussion. How are FAOSTAT prices for these crops determined. Are they farmgate prices or market prices that include high transport margins?

**Response**: The set of crop prices used in the Parity Model are based on FAO producer prices, which in principle are farmgate prices but in practice are often recorded from wholesale markets. FAO uses the Geary-Khamis method to derive, from national producer prices observed over 2004-2006, a set of average global commodity prices in purchasing-power-parity dollars per metric ton. FAO uses these prices to construct its measure of “Gross Production Value” in constant 2004-2006 international dollars for every crop in every country. We back out these prices by dividing FAO Gross Production Value by total quantity produced in a year. An advantage of using these FAO-derived prices is that they are an average based on a large set of producer prices from around the world. Moreover, it means that results can be easily reproduced and verified. This explanation has been added to the text. [*at line 156 in the revised document with track-changes*]

2. No distinction is made between food and feed uses of crops. Clearly, feed uses contribute less to energy and would favor richer consumers.

**Response**: Producer value is based on prices received for a commodity, regardless of its final use. Consumer value is based on what is actually consumed. Consumers gain in welfare if the prices they pay for food items decline, which will reflect the use to which commodities are put. Thus, if accelerated crop productivity leads to a decline in the price of grain, it is likely to have a large effect on the price of food grains and a small effect on the price of meat and milk (depending on the cost share of feed in total animal production costs). This in turn will affect quantities consumed, with larger effects on caloric consumption resulting from food uses than feed uses of these crop commodities. Measures of average caloric consumption change thus take into account final use of these commodities.

3. The analysis is for the public sector only. We know that for some crops, maize and potatoes, for example, private R&D is important and growing.

**Response**: We added a comment noting the role of private as well as public R&D in the Discussion section (on Implications: methods). [*at line 677*]

4. Table 7 on rankings deserves more discussion and sharper conclusions. Rice is clearly the number one priority in all cases. Wheat is also ranked high under most assumptions. At the same time, some crops are clearly low priority under all assumptions—barley and several of the legumes. The conclusions should clearly state these outliers and also that more data and more in depth analysis would be unlikely to change these conclusions.

**Response**: We added a sentence in the discussion of Table 7 noting that some crops are not highly ranked for any of the metrics or regions examined in this study, but might be ranked more highly for different criteria, locations, or populations groups. [*at line 548*]

Other comments
44. Needs to specify the base year

**Response**: This first sentence of the paper is a broad introductory statement noting that a wide range of studies (with different base years early in the 21^st^ century) support the conclusion that demand will rise significantly by mid-century. We feel it would be unnecessary and distracting to go into further detail on this point.

138. this is a big assumption and deserves more attention in the discussion.

**Response**: We have added to the text the following paragraph to explain and justify this assumption: “While a precise analysis of the welfare effects is not possible given the data available, we can draw some general conclusions by looking at the extent of poverty in the countries where significant economic impact was achieved. We premise this approach on the assumption that the economic benefits from accelerated productivity growth in crop staples are widely shared across income classes within a country. Technology adoption raises incomes of farm adopters and, through market-level effects, reduces prices paid by consumers for food. The crops affected are produced predominantly by smallholders, who tend to cluster at the lower end of the income scale. For consumers, income elasticities for these food staples are likely to be positive but small, meaning that per capita levels of consumption do not vary much across income classes (and, as a percentage of total expenditure, are higher for poor households). Thus it is reasonable to assume that benefits are roughly evenly distributed across the income strata of a country, and therefore the share of benefits accruing to those below a poverty line will be correlated with the poverty headcount index for that country at the time these impacts occurred.” [*at line 140*]

Table 2. No rationale is given for the selection of countries. China and Brazil are not included but India is.

**Response**: The purpose of the study was to identity research priorities for CGIAR crop improvement programs, which have historically focused on tropical and sub-tropical developing countries with limited capacities in agricultural research. In consultation with CGIAR donors we decided to exclude Brazil and China because they are both large, upper-middle-income countries. Southern Cone countries (Chile, Argentina, Paraguay and Uruguay) are also middle- or high-income countries that lie mostly in temperate areas, and therefore not a primary focus of the CGIAR. This explanation has been added to the text. [*at line 99*]

**Reviewer #2**:

The authors conduct an interesting analysis about priorities and benefits of agricultural productivity enhancements. There are many things I like about the analysis. However, the following comments will focus on potential weaknesses or shortcomings. I spend about 3 hours reading the paper. If I misinterpret some things, the authors should not hesitate to correct me.
**Response**: We thank the reviewer for this overall assessment. Specific comments are addressed below.

My main criticism is that the analysis seems somewhat incomplete and inconsistent.

1) The link between IMPACT and GLOBE seems to be a single feedback loop. I did not see a statement that this suffices to achieve consistency between the two models. It would find it sensible to iterate information between the two models until consistency is achieved. I don't think that it would take a lot of iterations but it would be useful to have some information about this in an appendix. Especially in poorer countries where agriculture contributes substantially to GDP, a single feedback may still leave biases.

**Response**: We realize that the use of the term ‘iteration’ in the original text is misleading, and have re-phrased and extended the text to clarify the nature of the GLOBE-IMPACT linkage (one-way from IMPACT to GLOBE at the dynamic baseline calibration stage, and one-way from GLOBE to IMPACT at the productivity improvement scenario simulation stage). See Delzeit et al. 2020 (reference added) for the distinction between one-way and two-way linkage approaches in the construction of baseline scenarios with linked models. [*at lines 241 and 267 in the revised document with track-changes*]

2) Parity analysis seems to provide only a demand value for productivity improvements. However, the cost of productivity improvements may differ greatly. For example, if a crop is not managed well in a certain region, parity analysis will suggest a lower priority (resulting from lower production quantities). However, the cost of productivity improvement may be much lower for low-yield situations (large yield gaps) than for crops which are already well managed and closer to some natural limits. The authors state clearly that they do not look into the cost of productivity improvements. However, I think they should at least discuss the issue of the yield gap a little bit better to avoid a misinterpretation of their results. Again, all other things equal, lower existing yields would with the applied method result in lower research priorities. I think, the lower existing yields (higher yield gaps) increase the attractiveness for research spending.

**Response**: We thank the reviewer for flagging this important point. We have added text in the Discussion section (under “Methods: indicators” and “Implications: methods” to note this limitation in our current analysis. [*at lines 641 and 680-682*]

To remedy this, the authors could use two alternative accounting schemes: a) price x area -> this switches off the yield impact, b) price x area x yield gap -> this would give priority to crops with higher yield gaps. The yield gap, ideally, should be calculated taking into account the regional distribution of soil-climate conditions. I believe there are already several studies which estimate regionally resolved yield gaps at global level.

**Response**: We thank the reviewer for these suggestions. They are beyond the scope of the present analysis, but we will make note of them for future work.

3) The authors estimate research spending priorities based on a single independent experiment for each crop where they raise the productivity of this crop by 25% (I believe globally). It would be very interesting to see what happens when a certain research budget is allocated simultaneously to regions and crops according to the estimated priorities. This would truly make the analysis complete and would consider the interactions between the crops. Then, they could trace out a response function where regionally diverse benefits of productivity increases are estimated against the regionally and crop specific expenses for different levels of budget. This, in my opinion would make the analysis really useful to policy makers.

**Response**: We agree this would be extremely useful. Two of our concluding points relate to the importance of improved data that would make this possible – including on the costs of technology development and dissemination and on its effectiveness in increasing productivity. We hope to be able to do this kind of analysis in the future.

Other comments

The poverty weighting schemes are good in principal but a bit crude. The crudeness comes from using the 1.90 $/day value as a black and white discrimination between poor and not poor. I would find it more sensible, if the authors would use an integral under an income distribution function. However, this would then also need additional assumptions on how to weight levels of poverty.
**Response**: We agree this would be interesting, but beyond the scope of the present analysis.

The authors state that the results data will be freely available and accessible. However, if I understand the policy of the journal correctly, they should also disclose the input data of the model.
Where is the information referred to in these statements:
".. baseline rates of productivity growth assumed in the IMPACT"
"IMPACT uses assumptions about key drivers such as population, income, technology, policy and climate to simulate changes in agricultural demand, production and markets for 60 commodities in 158 countries"

**Response**: The baseline productivity growth rates assumed in the model were developed in consultation with experts from each of the 15 CGIAR centers, and will be made available on GitHub. The socioeconomic and climate assumptions used in this analysis are drawn from the IPCC’s Shared Socioeconomic Pathway 2 and Representative Concentration Pathway 8.5, as noted in the second paragraph of the section on “Analysis using the IMPACT model”. More technical detail, including model equations, are available in Robinson et al. (2015) and the other references cited in this section.

**Reviewer #3**:

This paper is a follow-up of a study carried out by IFPRI and USDA-ERS for USAID to inform the Crops to End Hunger (CtEH) initiative. The latter is a development aid program which seeks to modernize public plant breeding in lower-income countries. The IFPRI-ERS study was aimed at assessing the impacts of faster productivity growth for selected food crops on income and other key indicators in developing countries in 2030.
The paper describes the method used and the main findings of this study. The method used is not original: this is a simulation exercise of a +25% increase of crop yields (crop by crop) in developing countries, carried out with the IMPACT model coupled with the GLOBE model (this coupling is already used in Mason-D’Croz et al., 2019, World Development); outputs are the impacts on economic surplus, prevalence of hunger and availability and adequacy of key nutrients (like in Nelson et al., 2018, Nature Sustainability). Results are unsurprising: as the yield increase applied successively to each crop is of the same magnitude (+25% relative to the baseline), the most important crops in terms of production value induce the strongest effects in terms of income and the most important crops in the diets induce the highest effects on food and nutritional security.
The paper is well-written and reports a competent work. But, I wonder what is the actual value added of the paper. Linked to this question, I have several concerns that I describe below.

**Response**: We thank the reviewer for this overall assessment. Regarding the question about value added, we make no claim to originality of approach. The paper describes an application of existing tools and methods to address specific questions from a development partner. We seek to publish this paper in *PLOS ONE* as a matter of record, transparency, and accessibility, noting that *PLOS ONE* evaluates research on “scientific validity, strong methodology, and high ethical standards—not perceived significance” (<https://journals.plos.org/plosone/static/publish>). Regarding the reviewer’s other concerns, please see our point-by-point responses below.

1. Major issues
i) I don’t understand why the authors refer to the parity model. This is very confusing and, as far as I can understand, it adds nothing to the analysis.

It is very confusing because, as mentioned by the authors, the parity model is commonly used to allocate R&D resources in multi-commodity systems. So initially the reader may imagine that the budget of the CtEH is allocated among the different crops in a first stage, that, in the second stage, the study estimates the impacts of additional R&D efforts on each crop yield and that, in the last stage, the study examines the impact of the induced differentiated yield changes in terms of income and food and nutritional security. In fact the study does not do that at all and the starting point is not the parity model but the assumed +25% yield increase for all crops. This +25% yield increase is simulated crop by crop and the induced impact in terms of income and food and nutritional security indicators are compared and analysed. Maybe I’ve missed something but in such approach, I don’t see what the parity model actually adds.

**Response**: The advantage of the parity model (also sometimes referred to as the “congruence model”) is that it is simple, intuitive, and easily to reproduce and verify. It is also a concept that is widely applied for resource allocation decision purposes in both public and private agricultural research programs. We extend the analysis beyond the parity model with the IMPACT economic model to capture important economic dimensions, such as how income and price changes affect consumer demand and commodity utilization. We also extend the analysis to include other impacts beyond income changes, such as poverty and nutrition outcomes. The fact that results are broadly consistent between the two approaches lends credence to the use of the parity model in decision making. We have added a note in the section on “Scenarios of faster productivity growth” to clarify that the productivity simulations are a separate and complementary approach. [*at line 198 in the revised document with track-changes*]

We can imagine that the parity model analysis could indicate which crops should receive higher shares of R&D resources. Then the simulation results would inform on which crops induce the highest income and food and nutritional effects following a x% increase in its yield. And finally we could examine whether the most efficient crops (in terms of increasing income and food and nutritional security) are also the ones which should receive higher shares of R&D resources according to the parity model (using alternative rules through poverty weights). But, once again as far as I understand, this is not was is done neither, otherwise authors would not state that “The scenarios of accelerated productivity growth reported in this note assume that investment in new varieties and other sources of on-farm productivity growth will increase sufficiently to result in a 25% increase in the annual rate of yield growth above “baseline” yield growth in farmers’ fields over the period 2015-2030” (li 77 to 80) or “A third departure from the simple parity model is to determine how accelerated crop yield growth might affect future incomes” (li 155-156).

Once again this is very confusing: are we in the first approach (impacts of R&D on yields, and impacts of yield changes on income and food and nutrition security), but in this case why a uniform +25% yield increase? Or are we in the second approach (impacts of yield changes on income and food and nutritional security and comparison with recommandations of R&D resource allocation according to the parity model), but in this case why justifying the 25% yield increase as resulting from additional R&D resources?

**Response**: Sorry for the confusion. We are using the second approach. We note that the hypothetical 25% increase in yields could result from additional investments in R&D and other sources of on-farm productivity growth, but we don’t analyze the sources of those yield increases, and they are not linked to the results of the parity analysis. We have revised the text to clarify this in the section on “Approach.” [*at line 86*]

ii) If the +25% yield increase results from additional R&D resources (from theCtEH I guess?), the paper should explain what are the bases of this assumption (empirical, literature, expert knowledge?). This is not done at all in the paper (which makes me think that we are rather in the second approach which does not require to explain the retained level of yield increase. This level could be 1%, 10%, 20%, etc., applying the same percentage to all crops being the only requirement in this case).

**Response**: Correct – we are using the second approach, and the yield increases are assumed to be of an arbitrary uniform percentage change for all crops to facilitate comparison.

iii) If I can understand that the authors use poverty rates to weight production values in the parity model I don’t understand why the same weights are applied to the simulated economic surplus from the accelerated productivity growth scenarios.

Once again if we are in the second approach, simulation results should be used as an estimation of the relative efficiency of each crop in increasing income and food and nutritional security and then should be compared to recommendations resulting from the parity model. The use of poverty weights makes sense in the parity model since it allows to show how the R&D resource allocation should change if we want to put emphasis on the poorest population. But, as far as I can understand, applying poverty weights to the simulation results of the accelerated productivity growth scenarios makes no sense in this case.

**Response**: Both the parity model and the simulation results provide estimates of income changes by country resulting from accelerated crop yield improvement: The parity model simply assumes these are likely to be significantly correlated with current value of crop production; the simulation model gives an explicit value of welfare impacts taking into account how changes in commodity prices affect consumer demand and commodity utilization. The purpose of the poverty weights is to give greater importance to income improvement among poor populations in poor countries. A data limitation is that we do not have estimates of future poverty rates, so our best approximation is to assume present poverty rates are likely to persist for the next decade or so. The manuscript has been amended to note this assumption and reasoning. [*at line 212*]

iv) Choosing clearly between the first or the second approach would provide a more clear grid for analysing results and highlighting the main findings, which is not the case currently.
Currently the analysis of scenarios’ simulation results are a mix between what it would be under the first option and what it would be under the second option. As a result, it is very difficult for the reader to see what these results really add.

**Response**: We have clarified that we are following the second approach. One of the reasons for using both parity analysis and productivity scenarios was to explore how the ranking of crops varies depending on the approach used and indicators chosen.

Minor issues
i) Introduction
- The paper should be situated relative to the existing literature and its originality, contribution and value added regarding existing knowledge should be described.

**Response**: We have added several references as suggested in the next comment below. Beyond that, we feel the references already included are sufficient for the purposes of this paper (which, as noted above, are to apply existing methods and tools to address specific questions from a development partner).

- Li 48 to 51. Changing diets and reducing food waste have also been advocated as part of the solution. This should be mentioned and related literature added.

**Response**: We have noted these additional strategies in the Introduction, along with several key references. [*at line 52*]

- Li 65-66: “This paper briefly describes how we did that and what we found”: this looks like a summary of a study report not like a research paper. This relates to the above-described major issues: what is the research question authors are dealing with? What do they want to show? This should be specified.

**Response**: We have deleted the sentence in question. The preceding sentence states the research question and purpose of the paper: to estimate the impacts of faster productivity growth for selected food crops on income and other key indicators in developing countries in 2030, in order to inform decision-making about the Crops to End Hunger initiative. [*at line 70*]

ii) Approach
- It should be made clear for what purpose the parity model is used (see major issues, first or second approach).

**Response**: We have revised the text to clarify this point in the “Approach” section and in the section on “Scenarios of faster productivity growth”. [*at lines 83 and 198*]

- Li 77-80: It should be made clear why the authors retained scenarios involving a uniform +25% yield increase? For what purpose such scenarios have been defined?

**Response**: Based on discussions with the donor agency, and in the absence of data on the costs of increasing yields for all target crops and countries, we ran scenarios assuming a hypothetical increase of 25% in the rate of productivity growth for each crop to facilitate comparison, across crops and countries, of the impacts of faster productivity growth. We have revised the text in the sections on “Approach” and “Scenarios of faster productivity growth” to clarify this. [*at Soc lines 83 and 198*]

iii) Analysis using the parity model
- Li 127-128: Explain why production quantities averaged over 2014-2016 are valued at international prices averaged over 2004-2006.

**Response**: FAO uses the Geary-Khamis method to derive, from national producer prices observed over 2004-2006, a set of average global commodity prices in purchasing-power-parity dollars per metric ton. FAO uses these prices to construct its measure of “Gross Production Value” in constant 2004-2006 international dollars for every crop in every country. An advantage of using these FAO-derived prices is that they are an average based on a large set of producer prices from around the world. Moreover, it means that results can be easily reproduced and verified. Further, no such internationally comparable average producer prices exist for a more recent period. This explanation has been added to the text. [*at line 156*]

iv) Scenarios of faster productivity growth
- Li 155-156: I don’t understand this sentence.

**Response**: This sentence simply introduces the use of scenarios to explore the future impacts of faster productivity growth. We have revised the text to clarify. [*at line 190*]

- Explain the 25% level of yield increase. Explain whether there is a link between these +25% and additional R&D resources from the CtEH program. Explain whether there is a link between these +25% and the parity model. The current version of the text is very confusing and it is difficult to understand where the +25% are coming from and what are they dedicated to show?

**Response**: We have clarified the text in various places, as noted above. We hope this is now clear.

v) Analysis using the IMPACT model
- Li 198-202: the iteration process between IMPACT and GLOBE should be described in more details. Only one iteration is mentioned: from IMPACT to GLOBE and then from GLOBE to IMPACT. Does it mean that only one iteration is sufficient to reach a consistent global equilibrium in both models?

**Response**: We realize that the use of the term ‘iteration’ in the original text is misleading, and have re-phrased and extended the text to clarify the nature of the GLOBE-IMPACT linkage (one-way from IMPACT to GLOBE at the dynamic baseline calibration stage, and one-way from GLOBE to IMPACT at the productivity improvement scenario simulation stage). See Delzeit et al. 2020 (reference added) for the distinction between one-way and two-way linkage approaches in the construction of baseline scenarios with linked models. [*at lines 241 and 267*]

vi) Analysis using the GLOBE model
- Li 222-225: the way the “agricultural productivity enhancement from the various scenarios … in IMPACT” are translated into “shifts of the factor productivity parameters in the agricultural production functions” should be described in more details. Are these productivity shifts also affecting land rents (only wages and capital returns are mentioned)?

**Response**: Yes, they are. We have clarified the text accordingly. [*at line 276*]

vii) Results
- Li 348-350: “Growth in unweighted economy-wide income was largest in South Asia, but when weighted by the poverty gap, the largest increase occurred in Sub-Saharan Africa.”. Maybe “occurred” is not the right verb.

**Response**: We have revised the wording to say “was estimated to have occurred”. [*at line 403*]

- Table 4a: instead of totals computed as the sum of the values obtained from each crop scenario, a “true” total reporting the effect of a +25% yield increase for all crops considered simultaneously would bring additional information on the interaction between crop markets and cross effects of additional R&D resources allocated to various crops.

**Response**: This would be a valuable addition to consider for future work.

- Table 4c: Could be moved to additional information or appendix.

**Response**: Since the journal does not impose a page limit, we have decided to keep the table in its current location.

**Journal Requirements**:

When submitting your revision, we need you to address these additional requirements.

1. Please ensure that your manuscript meets PLOS ONE's style requirements, including those for file naming. The PLOS ONE style templates can be found at

<https://journals.plos.org/plosone/s/file?id=wjVg/PLOSOne_formatting_sample_main_body.pdf> and

<https://journals.plos.org/plosone/s/file?id=ba62/PLOSOne_formatting_sample_title_authors_affiliations.pdf>

**Response**: Done.

2. We note that you have stated that you will provide repository information for your data at acceptance. Should your manuscript be accepted for publication, we will hold it until you provide the relevant accession numbers or DOIs necessary to access your data. If you wish to make changes to your Data Availability statement, please describe these changes in your cover letter and we will update your Data Availability statement to reflect the information you provide.

**Response**: We will upload the data to GitHub and provide the relevant access information prior to publication.

3. We note that Figures 2, 3a, 3b and 4 in your submission contain map images which may be copyrighted. All PLOS content is published under the Creative Commons Attribution License (CC BY 4.0), which means that the manuscript, images, and Supporting Information files will be freely available online, and any third party is permitted to access, download, copy, distribute, and use these materials in any way, even commercially, with proper attribution. For these reasons, we cannot publish previously copyrighted maps or satellite images created using proprietary data, such as Google software (Google Maps, Street View, and Earth). For more information, see our copyright guidelines: <http://journals.plos.org/plosone/s/licenses-and-copyright>.

We require you to either (1) present written permission from the copyright holder to publish these figures specifically under the CC BY 4.0 license, or (2) remove the figures from your submission:

3.1.    You may seek permission from the original copyright holder of Figures 2, 3a, 3b and 4 to publish the content specifically under the CC BY 4.0 license.

We recommend that you contact the original copyright holder with the Content Permission Form (<http://journals.plos.org/plosone/s/file?id=7c09/content-permission-form.pdf>) and the following text:

“I request permission for the open-access journal PLOS ONE to publish XXX under the Creative Commons Attribution License (CCAL) CC BY 4.0 (<http://creativecommons.org/licenses/by/4.0/>). Please be aware that this license allows unrestricted use and distribution, even commercially, by third parties. Please reply and provide explicit written permission to publish XXX under a CC BY license and complete the attached form.”

Please upload the completed Content Permission Form or other proof of granted permissions as an "Other" file with your submission.

In the figure caption of the copyrighted figure, please include the following text: “Reprinted from [ref] under a CC BY license, with permission from [name of publisher], original copyright [original copyright year].”

3.2.    If you are unable to obtain permission from the original copyright holder to publish these figures under the CC BY 4.0 license or if the copyright holder’s requirements are incompatible with the CC BY 4.0 license, please either i) remove the figure or ii) supply a replacement figure that complies with the CC BY 4.0 license. Please check copyright information on all replacement figures and update the figure caption with source information. If applicable, please specify in the figure caption text when a figure is similar but not identical to the original image and is therefore for illustrative purposes only.

The following resources for replacing copyrighted map figures may be helpful:

USGS National Map Viewer (public domain): <http://viewer.nationalmap.gov/viewer/>

The Gateway to Astronaut Photography of Earth (public domain): <http://eol.jsc.nasa.gov/sseop/clickmap/>

Maps at the CIA (public domain): <https://www.cia.gov/library/publications/the-world-factbook/index.html> and <https://www.cia.gov/library/publications/cia-maps-publications/index.html>

NASA Earth Observatory (public domain): <http://earthobservatory.nasa.gov/>

Landsat: <http://landsat.visibleearth.nasa.gov/>

USGS EROS (Earth Resources Observatory and Science (EROS) Center) (public domain): [http://eros.usgs.gov/#](http://eros.usgs.gov/)

Natural Earth (public domain): <http://www.naturalearthdata.com/>

**Response**: We have added additional source information for the maps in figures 2, 3a, 3b and 4.

**Figure 2** uses an adapted world country boundary map in the ArcWorld Supplement from ESRI and created for display here using the free and open source QGIS. As explained at <https://www.esri.com/arcgis-blog/products/product/mapping/using-and-citing-esri-data/> and <http://help.arcgis.com/en/arcgisdesktop/10.0/help/index.html#//001z00000003000000.htm>, the world country boundary map is subject to the following statement: "Redistribution rights are granted by the data vendor for hardcopy renditions or static, electronic map images (for example, .gif, .jpeg) that are plotted, printed, or publicly displayed with proper metadata and source/copyright attribution to the respective data vendor/vendors." Using a fully licensed ERSI product containing the ArcWorld Supplement allows for use of the adapted base map in academic publications as used here.

**Figures 3a, 3b and 4** use Natural Earth map files. As stated at <https://www.naturalearthdata.com/about/terms-of-use/>, “All versions of Natural Earth raster + vector map data found on this website are in the public domain. You may use the maps in any manner, including modifying the content and design, electronic dissemination, and offset printing... No permission is needed to use Natural Earth. Crediting the authors is unnecessary.”
