# Supplementary material for: Modeling impacts of faster productivity growth to inform the CGIAR initiative on Crops to End Hunger
Source: PLoS One. 2021 Apr 15;16(4):e0249994. doi: 10.1371/journal.pone.0249994 (PMC8049331; doi:10.1371/journal.pone.0249994)
Supplement: Attachment — Submitted filename: Response to reviewers-PONE-D-20-06988R1.docx [file pone.0249994.s002.docx]

**Response to reviewers of “Modeling impacts of faster crop productivity growth to inform the CGIAR initiative on Crops to End Hunger” [PONE-D-20-06988R1]**

by Keith Wiebe, Timothy B. Sulser, Shahnila Dunston, Mark W. Rosegrant, Keith Fuglie, Dirk Willenbockel, and Gerald C. Nelson

11 January 2021

**Authors’ responses** are highlighted in grey.

**Additional Editor Comments**:

The fundamental issue with any paper based on a modeling exercise that may be used beyond the bubble of modelers that know and understand the inner workings of a model is to provide sufficient context to allow the model outcome analysis results to be used in a meaningful way. The results presented in the paper will be variously used without necessarily a full understanding of the limitations.
This requires some modesty is presentation. It also requires providing clear delimitation of scope and related to that what key assumptions have been made at the model level and at the scenario level. The best way to do this is to provide adequate supplementary material spelling out explicitly assumptions, data sources, standards and definitions used.
Providing (a link to) the data used in the analysis is a prerequisite for publication in PLOS One anyway. Not all data are easily accessible.

**Response**: We believe the paper is sufficiently modest in presentation already, noting that the Discussion section (more than 5 pages) is devoted almost entirely to discussing caveats, limitations, and the need for further research. We have made the model parameter values and results available on GitHub at <https://github.com/IFPRI/IMPACT>. Scenario assumptions are spelled out clearly in the text.

There are numerous other papers using the same suite of models by largely the same set of authors. This paper potentially adds value but needs to be more transparent about some of the issues raised by the reviewers. In any case, the paper is already available in SocArXiv (<https://doi.org/10.31235/osf.io/h2g6r>) so this version when (/if) published should try to address remaining issues for the benefit of all.

**Response**: We thank the editor and reviewers for their additional comments on the revised version. We have addressed these comments as explained in our point-by-point responses below.

**Reviewer #3**:

Thank you for your point-by-point responses to my first round comments. I agree with most of your responses but I still have one concern that I detail below.

I think I’ve finally found why I had difficulties to connect the parity model approach and the IMPACT-GLOBE simulation approach proposed here: the production value share indicator used in the parity model approach is very different from the ES value share indicator used in the IMPACT-GLOBE simulation approach. And I even wonder if both indicators can be compared, from an economic point of view.
Therefore, I think the authors should explain the exact meaning of their ES (economic surplus) share indicator. When dealing with the value shares, we refer to an initial situation (FAO data 2015) or to a single scenario (IMPACT model 2015 or IMPACT model 2030 reference scenario). In that case, the value share of each crop is the production value of that crop divided by the sum of all crop production values in the same situation or scenario.
When dealing with the ES shares, the situation is very different. If I understand well the so-called Economic surplus (ES) of each crop (Tab. 4a) is the welfare change induced by the +25% productivity improvement simulated for that crop relative to the reference scenario. Thus the ES of each crop results from different scenarios (ES rice is the welfare change resulting from the rice productivity improvement scenario, the maize ES is the welfare change resulting from the maize productivity improvement scenario and so on). In such a case, what is the meaning of the sum of ES over all crops (the sum of the welfare changes obtained from the individual crop productivity improvement scenarios, which is clearly different from the welfare change resulting from the all crops productivity improvement scenario)? Furthermore, what is the meaning of the ES share of each crop? Once again, I am not sure I understood correctly, but the ES share of one crop is the welfare change resulting from that crop productivity improvement scenario divided by the sum of the welfare changes resulting from all individual crop productivity improvement scenarios. I am not sure I get exactly what does this ES share indicator mean. Is this indicator consistent from an economic point of view? Can it be compared with the production value share indicator used in the parity model? It would be consistent and comparable to the production value share indicator if the ES share of one crop was the welfare change resulting from that crop productivity improvement scenario divided by the welfare change resulting from the scenario involving simultaneous productivity improvement of all crops (the ES share of one crop would measure the contribution of that crop productivity improvement to the global welfare change obtained from the productivity enhancement of all crops).
Maybe I’ve missed something and I’ll be happy to get some explanation from the authors. But if I understood correctly, at least this point should be made clear to the reader and implications should be discussed.

**Response**: We have revised the text to clarify that the “ES Share” is a measure of the *relative* size of the welfare gain expected from productivity improvement in a crop. It simply converts the estimates of ES (in dollars) into an index that sums to 100, and as such provides a decision guide for how a research budget could be efficiently allocated across commodities. Further, this metric is directly comparable to the parity ratios derived from gross production values. Under the assumptions noted in the text, an efficient allocation of research expenditures (one that maximizes returns to research) would imply that the research intensity (the ratio of production value to research expenditure) for all commodities should be the same. In the absence of complete and consistent data on the current allocation of research, a parity ratio – the value share of a commodity in the total value of the commodities in question – provides equivalent information, so setting the research expenditure share equal to the value share would assure that the research intensities are equal.

**Reviewer #4**:

The revised paper has been variously updated in response to reviewer comments. Overall, this has improved and clarified a number of issues raised. Yet a number of issues remain. Given the complexity of the modeling and associated assumptions and implications of the results do see the need to address these issues clearly. Some readers may use the quantitative results at face value – especially as many will not understand or easily find the many underlying and sometimes substantial assumptions.
Some major issues:
1. Crop prices used: The paper uses “global average commodity prices from 2004-06 (i.e., in constant 2005 international dollars)”. Various reviewers took issue with these outdated prices and the authors did add but not resolve the issue. The paper and Tables value the different commodities using FAO 2014-16 production data and these 2004-06 average prices (a decade earlier!). Tables and scenarios always refer to “2015” (e.g. Table headings: “Gross production value from FAOSTAT in 2015”), yet using outdated prices which is then mentioned in the footnotes. This is not only confusing but also particularly problematic:
a. As prices are 1 of the 2 key factors determining the relative commodity values in the baseline year.
b. The world has had a global food crisis during this interlaying decade with prices for some of these food crops showing significant movements and being at the heart of the crisis. It seems rather simplistic to just use the outdated prices and assume that there may not have been any relative shift in prices since.
c. The models and scenarios subsequently project to 2030. For such 15 year projections one would expect to use indicators/trends as close to the 2015 base year as possible – and not add another decade of noise and potential bias with outdated prices …
The authors add some text (incl that FAO uses the Geary-Khamis method to derive a set of average global commodity prices in purchasing-power-parity dollars per metric ton) and refers to FAOStat . The text states “no such internationally comparable average producer prices exist for a more recent period” – yet the referred to FAOStat does includes more recent price data. Further effort should be made to really use 2015 as a base year for projections – not as an ambiguous and confounding base year that combines prices for a decade earlier (2004-06!) with production data from 2015.

**Response**: We have explained in the text that the advantage of using this set of prices is that they provide a standardized, revenue-weighted set of commodity prices, expressed in purchasing-power-parity dollars per ton, which value quantities of crops produced around the world in a consistent way. FAO derives this set of prices using the Geary-Khamis method applied to national producer prices from around the world and uses them to construct its index of “Gross Production Value” of every crop in every country in constant international dollars. (We back out these prices by dividing the FAO Gross Production Value by total quantity produced in a year). We used FAO prices for 2004-2006 because they were the latest available at the time the study was conducted, and they are also consistent with IMPACT’s base year of 2005. Although FAO recently updated its Gross Production Value index using prices from 2014-2016, redoing the analysis with updated prices would have little effect on the relative value of production or productivity changes across crops, because a comparison of 2014-2016 prices with 2004-2006 prices reveals that relative crop prices – the value of a ton of wheat relative to a ton of beans, for example – remained quite stable between the two periods despite fluctuations in some years. Thus, while using more recent prices would shift the nominal value of all crops upward, it would have little or no effect on relative values of production or productivity changes across crops, determination of which is the primary objective of this study.

2. Clarifying critical assumptions in additional tables: One could argue over a number of the underlying assumptions. But much of the models remain a black box – with limited insight in what is actually happening. In the responses authors mention uploading the data to GitHub and providing the relevant access information prior to publication. But one would expect to at least be able to access some of the critical assumptions before deciding on publication and the utility of the paper – also for subsequent users. The paper is pretty long – but it would not hurt to add a few tables with the critical assumptions as they apply to each of the studied crops – in the supplementary data section if preferred. The authors do variously mention the intention to improve on these results in the discussion – why not then at least share the underlying key assumptions …? For instance:
a. Baseline productivity growth rates assumed in the IMPACT model: The paper states the authors use these base rates and then increase these with a flat 25% and project to 2030. In the current version these basic historic rates are a key driver for 2030 impacts and would be good to clarify and make these underlying rates clearly accessible or point to full details if available elsewhere … Now such statements on p. 34 as “Baseline productivity growth rates in IMPACT are based on the principles laid out” in the literature. “These have been subsequently adjusted based on expert opinion and in consultations”. “However, there is always room for improvement”. If the underlying growth rate was negligible – 25% increase may not do much. Please just be specific and transparent and list them out.
b. p. 34-5 “Demand elasticities are important”. “Alternative elasticities would lead to different outcomes, but again, relative rankings are likely to be similar across the plausible range of elasticities.” Again, please just be specific and transparent and list them out.

**Response**: We have made the productivity and elasticity parameters available online at <https://github.com/IFPRI/IMPACT>. Further information on these parameters and their sources is available in Robinson et al. (2015) (reference [19] in the present paper).

3. Avoiding ambiguous indicators:
a. Clarifying the base reference: In a number of instances the 2030 implications are presented as changes relative to a base – but no actual base reference numbers are provided to at least gauge the relative magnitude. A clear example is Table 5. The proposed scenarios would lift a potential aggregate of 27.5M out of hunger relative to the “reference scenario in 2030”. So what was the projected hunger rate in 2030 as well as the current/base rate? Same for undernourished children.

**Response**: Our focus is primarily on changes relative to a reference scenario. For example, Table 5 explicitly presents both percentage and absolute changes relative to the reference scenario in 2030, so readers can directly see the relative magnitude of the change (and also calculate the reference value if they wish).

b. “Adequacy ratios”: Fig 3 presents adequacy ratios for the reference case in 2030 for selected nutrients and vitamins. For a number of countries some adequacy ratios exceed 3, and for carbohydrates this is nearly global. This seems high vis-à-vis concerns of feeding the world. It also contradicts earlier published findings by many of the same authors using the same underlying models. E.g. Mason-D’Croz et al., 2019, World Development Fig 8 depicts average food supply (kilocalorie per person per day) in 2010 and 2030 with global average supply typically approaching the recommended daily consumption of an active 20 to 35-year-old male. Please cross-check what is going on and update as needed. Suggest to drop Fig 3.

**Response**: Thanks for flagging this issue. Part of the confusion here comes from the distinction between adequacy of total caloric availability (as reported by Mason-D’Croz et al. (2019 *World Development*) and adequacy of particular nutrients (as discussed here). Since there are three primary sources of caloric intake in a diet – carbohydrates (including starches and sugars, and alcoholic beverages in some countries), fats, and protein, it is possible for an individual to consume amounts of carbohydrates well above RDA levels and still have a shortfall of total caloric intake. (For example, the average daily Kcal availability in Mexico is around 3200, about 60 percent of which are from carbohydrates, indicating a carbohydrate adequacy ratio of about 4.) We have made that distinction more explicit in the text. An extended discussion of the adequacy ratio and the methodology to estimate it is available in the supplementary material of Nelson et al. (2018 *Nature Sustainability*) (reference [45] in the present paper). Additional graphics and numeric details are available at <https://impactnutrients.ifpri.org/nutrientModeling/>.

c. “dietary diversity”: p. 30 states “Several measures of diversity are available. For this report, we use the non-staple share of energy intake.” This is a rather questionable choice. Dietary diversity primarily relates to diverse food groups to ensure adequate intake across nutrients. Staples are a good and generally cheaper source of energy – diversity is needed to ensure adequacy in relation to other nutrients. Also questions arise around what the authors define as “staples”. E.g. surprised to note that banana’s are not considered staples: “The only yield increases that raise the non-staple share more than 0.01 percent are for groundnuts in SSA (0.08 percent) and banana in Asia and SSA (0.03 and 0.04 respectively)”. Also questions about what is actually included in the “nonstaple” share of kCals in much of the Global North (>60%). Drop Fig 4 given this ambiguity.

**Response**: The non-staple share of energy intake is widely used as a single diversity measure in the nutrition literature. For example, see Lele et al. (2016), Measuring Food and Nutrition Security: An Independent Technical Assessment and User’s Guide for Existing Indicators. We have added this reference to the text. We have also explained that in this analysis, bananas are considered a non-staple, while plantains are considered a staple. Further information on alternative diversity indicators (Shannon Diversity and Rao’s Quadratic Entropy) as well as details on what are considered staples and non-staples can be found at <https://impactnutrients.ifpri.org/nutrientModeling/>.

d. Fig 5 has a rather unclear legend for the metrics presented as they represent different years (2015, 2030) and scope (some seemingly share in the total metric for the year – others like undernourished children and hunger share in the change of the metric?). Crop value clearly relates to 2 different years/estimates; but ES – to which year does this relate (2030?). Also reference to “shocks” in the title – whereas ones assumes this relates to the assumed 25% productivity increase for each crop? Table 7 – same issue with metrics as Fig 5.

**Response**: Thanks for flagging this issue. We have clarified the titles and legends of Figure 5 and Table 7 accordingly.
