# Supplementary material for: Modeling impacts of faster productivity growth to inform the CGIAR initiative on Crops to End Hunger
Source: PLoS One. 2021 Apr 15;16(4):e0249994. doi: 10.1371/journal.pone.0249994 (PMC8049331; doi:10.1371/journal.pone.0249994)
Supplement: Attachment — Submitted filename: Response to reviewers-PONE-D-20-06988R2.docx [file pone.0249994.s003.docx]

**Response to reviewers of “Modeling impacts of faster crop productivity growth to inform the CGIAR initiative on Crops to End Hunger” [PONE-D-20-06988R2]**

by Keith Wiebe, Timothy B. Sulser, Shahnila Dunston, Mark W. Rosegrant, Keith Fuglie, Dirk Willenbockel, and Gerald C. Nelson

26 March 2021

**Authors’ responses** are highlighted in grey.

We thank the editor and reviewers for their additional comments on the revised version. We have addressed these comments as explained in our point-by-point responses below.

**Reviewer #3**:

I think the explanation added as a note to Table 4 (a, b and c)( for ease of comparison ...) is not useful and may be confusing. What I requested was a clear explanation that ESS=ES share = ES cropi/Sum over cropi (EScropi) and that EScropi is a different scenario for each cropi. So that ES crop1 is Economic surplus for scenario1 and ES share crop1= ES scenario1/ES scenario1+ES scenario2+ES scenario3+...
In addition if you decide to keep the sentence "For ease of comparison ..." It should come after the definition of ESS. This is not the case currently..

**Response**: We have clarified the notes to Tables 4a, 4b and 4c accordingly.

**Additional Editor Comments**:

A word of caution is warranted about the data used that may pose some problems. This was raised earlier in the review process as well. two examples stand out:

1. weighing with poverty rates is not completely unproblematic, when this data is missing for some countries.
2. Prices for roots and tubers sometimes refer to fresh produce and sometimes to dried commodities. For instance for cassava in west Africa this is an issue.

Addressing these data issues always has an arbitrary component to it. That is not a problem as long as you are transparent about this.

1. Reviewer number 3 has a valid point about the note with Table 4. Please clarify.

**Response**: We have clarified the notes to Tables 4a, 4b and 4c as requested by Reviewer 3.

2. Value based analyses depend critically on prices used. Prices are notoriously tricky values in global statistics because there is not always clarity about what product the price refers to. For instance roots and tubers are harvested with high moisture content reflected in production statistics, prices sometimes refer to the wet product and sometimes to dried product. The overly high values of for instance cassava in west Africa can be attributed to this issue. Without delving into details, a note of caution about values is warranted. The Geary-Khamis method does not solve this. This implies a word of caution about the statement in lines 390-393.

**Response**: Prices are measured at the farm-gate or rural wholesale level in terms of fresh weight (not dry weight) for all commodities in dollars per metric ton. (One of the authors has 10 years of prior experience with CIP in Africa and Asia and has looked into this issue in the past, for example, by comparing the country-level prices reported by FAO for these commodities with IITA and other cassava marketing studies from Nigeria and Ghana. He found that the prices reported by FAO are generally consistent with what marketing reports show for wholesale fresh root prices.) While the quality of price information will naturally vary, by taking a global average over a three-year period, FAO attempts to minimize distortions that may emerge from poor quality data in some countries. We have clarified these points at lines 154 and 161. Since these points about price measurement and data quality apply to all values, we did not add a further comment at lines 390-393.

3. The weighing scheme based on $1.90/day/capita poverty rate [24] see line 173 assumes comparable data. Not all countries are covered and the data for different countries come from different years. A note of caution is therefore warranted besides the warning in line 221.

**Response**: We have noted at line 179 that data coverage varies across countries and years.

**Figures**

While revising your submission, please upload your figure files to the Preflight Analysis and Conversion Engine (PACE) digital diagnostic tool, <https://pacev2.apexcovantage.com/>. PACE helps ensure that figures meet PLOS requirements. To use PACE, you must first register as a user. Registration is free. Then, login and navigate to the UPLOAD tab, where you will find detailed instructions on how to use the tool. If you encounter any issues or have any questions when using PACE, please email PLOS at [figures@plos.org](mailto:figures@plos.org). Please note that Supporting Information files do not need this step.

**Response**: We have uploaded our figures to the PACE diagnostic tool and submitted the resulting versions.
